# Impact on *Aedes aegypti* Mosquitoes Exposed to Honey-Impregnated Flinders Technology Associates (FTA^®^) Cards

**DOI:** 10.3390/tropicalmed9070165

**Published:** 2024-07-20

**Authors:** Amandine Guidez, Albin Fontaine, Arnaud Cannet, Isabelle Dusfour, Romain Girod, Sébastien Briolant

**Affiliations:** 1 Unité d’Entomologie Médicale, Institut Pasteur de la Guyane, 97306 Cayenne, France; idusfour@gmail.com (I.D.); romain.girod@pasteur.fr (R.G.); 2 Unité des Virus Émergents, Aix-Marseille University, Università di Corsica, IRD 190, Inserm 1207, IRBA, 13005 Marseille, France; albinfont@gmail.com; 3 Unité de Virologie, Institut de Recherche Biomédicale des Armées (IRBA), 13005 Marseille, France; 4 CNEV, IRD, 34000 Montpellier, France; 5 Unité Parasitologie et Entomologie, Département de Microbiologie et Maladies Infectieuses, Institut de Recherche Biomédicale des Armées, 13005 Marseille, France; sbriolant@wanadoo.fr; 6 Aix Marseille Université, SSA, AP-HM, UMR Risques Infectieux Tropicaux et Microorganismes Emergents (RITMES), 13005 Marseille, France; 7 IHU Méditerranée Infection, 13005 Marseille, France

**Keywords:** arboviruses, surveillance, *Aedes aegypti*, blotting paper, FTA^®^ cards, sugar feeding

## Abstract

Programs to control viruses transmitted by mosquitoes requires the implementation of surveillance tools. Over the past decade, Flinders Technology Associates (FTA^®^) cards, which preserve nucleic acids, have emerged as an innovating surveillance system for collecting arboviruses expectorated during mosquito sugar feeding. In this study, we evaluate the survival rates of two strain of *Aedes aegypti* (New Orleans (NO) and Cayenne (CAY)) in the laboratory after exposing to either honey-impregnated FTA^®^ cards or untreated filter paper (UFP) card. Experimental exposure of mosquitoes to FTA^®^ cards during sugar feeding significantly negatively impacted their survival, as compared to untreated filter paper. The median survival time was 2 days (95% confidence interval [CI] 1 day, 3 days) for mosquitoes exposed to FTA cards from strain NO and 3 days (95% CI 2 days, 3 days) for mosquitoes exposed to FTA cards from strain CAY. Mosquitoes exposed to UFP did not survive until the end of the experiment (4 days for strain NO and 7 days for strain CAY). Although this finding does not preclude the use of FTA^®^ cards in surveillance, it is crucial to consider and incorporate this factor into study designs.

The Flinders Technology Associates (FTA^®^, Whatman, GE Healthcare, Florham Park, NJ, USA) cards are used for the collection, storage, transport, and isolation of high-quality DNA or RNA biological sample for downstream applications [1]. FTA cards^®^ potentially provides a useful medium to help overcome the difficulties of maintaining viral integrity for pathogen detection, as they are a filter paper matrix mixed with a proprietary mixture of chemicals that lyse cells and stabilize nucleic acids on contact for long-term storage at room temperature. Initially used for the transport of human samples, FTA^®^ cards have also been used for the collection of saliva and mosquito excreta [2,3,4,5,6]. Infectious mosquitoes can expectorate viruses via their feces or saliva. The detection of virus in the saliva of mosquitoes indicates systemic infection and transmission capacity at the time of collection. In contrast, the presence of a virus in their excreta does not reflect their vector competence status [7]. FTA^®^ filter paper cards soaked in honey have been used in several studies and in the field to detect arboviruses expectorated in mosquito saliva during sugar feeding [4,5,6,8,9]. This technique has a favorable cost/efficiency ratio because it allows the virus to be detected in a community of trapped mosquitoes at once [4,6]. However, field observations describe a low survival rate in the traps [6,10]. The purpose of this study was to assess the impact on mosquito survival rate over time in the laboratory when exposed to honey-impregnated FTA^®^ cards as compared to simple honey-impregnated on untreated filter paper (UFP).

Laboratory experiments were conducted using two types of filter paper for comparison: FTA^®^ cards and UFP. Two strains of *Ae*. *aegypti* were used in the study: an established laboratory colony (Fx generation) from New Orleans (NO) and a wild-type strain (F1 generation) from Cayenne (CAY). Cards were placed in small plastic bags with an opening hole at their center and suspended from the top of a 110 mm high and 80 mm in diameter cardboard containers. The experiment was performed in triplicates with 30 mosquitoes exposed to either honey-impregnated UFP cards or to honey-impregnated FTA cards. We considered that all mosquitoes took sugar as a daily basis. Mosquitoes were maintained under controlled insectary conditions (28 ± 1 °C, 70 ± 10% relative humidity, and 12:12 h light–dark cycle). Mortality was monitored every day at the same time for all conditions.

Survival analysis was performed using the survival (v 3.2.10), and survminer (v 0.4.9) packages in the R statistical environment (v 4.3.2) [11,12,13]. A log-rank test was used to compare censored survival times in mosquitoes experimentally exposed to FTA^®^ cards and UFP (Figure 1). For the CAY strain, the median survival time was 3 days (95% confidence interval [CI]; from 2 days to 3 days) for mosquitoes exposed to FTA cards and was never reached by mosquitoes exposed to UFP until the end of the experiment (7 days). The probability to survive beyond 7 days was 4.31% (95% CI 1.45%, 12.82%) for mosquitoes exposed to FTA cards and 75.56% (95% CI 67.18%, 84.98%) for mosquitoes exposed to UFP. The card type used by mosquitoes to feed on sugar had a significant impact on survival (*p* < 0.0001, log-rank test). For the NO strain, the median survival time was 2 days (95% confidence interval [CI]; 1 day to 3 days) for mosquitoes exposed to FTA cards and 3 days (95% CI; 2 days to 3 days) for mosquitoes exposed to WM cards. No mosquitoes exposed to FTA cards survived beyond 7 days (95% CI 0%, 0%), whereas 43.33% (95% CI 25.17% to 64.08%) of mosquitoes exposed to WM cards survived beyond 7 days. The type of card used by mosquitoes for sugar feeding significantly influenced survival (*p* = 0.00042, log-rank test). Regardless of the mosquito strains tested, UFP cards is significantly more beneficial for mosquito survival than FTA^®^ cards.

An innovative surveillance system leverages the physiological phenomenon where infected mosquitoes expectorate the virus while feeding on honey, an attractive sugar source^9^. Many systems use FTA^®^ cards to hold the honey, as these cards preserve the nucleic acids. Additionally, honey can be mixed with a blue dye, causing the abdomens of feeding mosquitoes to turn blue, confirming their consumption of the sugar bait. Several field studies have captured blue-colored female and male mosquitoes and witness mortalities induced by FTA^®^ cards [6,14]. Here, we confirmed experimentally that filter papers treated to preserve DNA have shortened the life expectancy of mosquitoes. After one week of exposure to FTA^®^ cards, the CAY and NO strains exhibited no or less than 5% survival probability. Conversely, mosquitoes exposed to WM cards showed survival rates of 75% for the CAY strain and more than 65% for the NO strain at this time. Increasing trapped mosquito survival would directly optimize the yield of the primary biological resource (either saliva or excreta) and thus increase the sensitivity of detection when trapped mosquitoes are preserved alive on the field (usually from 24 h to 1 week). Further work would be needed to explore the balance between nucleic acid preservation and mosquito survival. Viral RNA was shown to be well preserved in dried blood spots on untreated filter papers over several weeks [3,15,16]. Alternatively, the insecticidal effect of FTA^®^ cards, without acting as a repellent, makes them suitable for use as attractive toxic sugar baits (ATSBs) for mosquito control [17]. The use of FTA^®^ cards for saliva collection must then be tailored to the purpose of the study.

In conclusion, FTA^®^ cards leverage the natural behavior of mosquitoes to expectorate viruses while feeding on honey, but they also significantly reduce mosquito survival rates compared to UFP. Our laboratory experiments show that less than 5% of mosquitoes exposed to FTA^®^ cards survive after one week, indicating a toxic effect. This high mortality can be advantageous for mosquito control, using FTA^®^ cards as attractive toxic sugar baits (ATSBs). However, the toxic effect poses challenges for mosquito surveillance programs, as it limits the collection of viable biological samples. Balancing nucleic acid preservation with mosquito survival is essential to optimize the use of FTA^®^ cards in surveillance efforts.

## Figures and Tables

**Figure 1:**
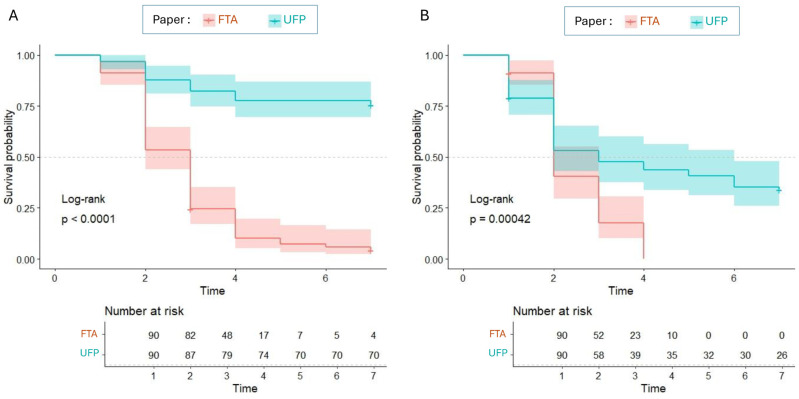
Kaplan–Meier curves representing survival probability estimates for mosquitoes orally exposed to FTA^®^ and UFP cards for sugar feeding. Survival curves and corresponding risk tables are shown by condition only with 95% confidence intervals in light shading for CAY strain (**A**) or NO strain (**B**). A log-rank test was used to compare survival times of mosquitoes experimentally exposed to FTA^®^ and UFP cards.

## Data Availability

Data Availability Statement: The data presented in this study are available on request from the corresponding author.
